# Social anxiety moderates the association between adolescent irritability and bully perpetration

**DOI:** 10.1017/S0954579424000439

**Published:** 2024-03-13

**Authors:** Michael T. Perino, Jennifer C. Harper-Lednicky, Alecia C. Vogel, Chad M. Sylvester, Deanna M. Barch, Joan L. Luby

**Affiliations:** 1 Department of Psychiatry, Washington University School of Medicine, St. Louis, MO, USA; 2 Department of Psychology and Brain Sciences, Washington University in St Louis, St. Louis, MO, USA

**Keywords:** Bully perpetration, irritability, social anxiety

## Abstract

**Background:**

Preliminary work suggests anxiety moderates the relationship between irritability and bullying. As anxiety increases, the link between irritability and perpetration decreases. We hypothesize that any moderation effect of anxiety is driven by social anxiety symptoms. We sought to explicate the moderating effect of anxiety, while clarifying relations to other aggressive behaviors.

**Methods:**

A sample of adolescents (*n* = 169, mean = 12.42 years of age) were assessed using clinician rated assessments of anxiety, parent reports of irritability and bullying behaviors (perpetration, generalized aggression, and victimization). Correlations assessed zero-order relations between variables, and regression-based moderation analyses were used to test interactions. Johnson–Neyman methods were used to represent significant interactions.

**Results:**

Irritability was significantly related to bullying (*r* = .403, *p* < .001). Social, but not generalized, anxiety symptoms significantly moderated the effect of irritability on bully perpetration (*t*(160) = −2.94, *b* = −.01, *p* = .0038, Δ*R*^2^ = .0229, *F*(1, 160) = 8.635). As social anxiety symptoms increase, the link between irritability and perpetration decreases.

**Conclusions:**

Understanding how psychopathology interacts with social behaviors is of great importance. Higher social anxiety is linked to reduced relations between irritability and bullying; however, the link between irritability and other aggression remains positive. Comprehensively assessing how treatment of psychopathology impacts social behaviors may improve future intervention.

## Introduction

Bullying in youth is a major societal and public health problem linked to significant psychological (Vaillancourt et al., 2013), economic (Wolke et al., 2013), and societal harms (Jantzer et al., 2019). Understood as aggressive social behaviors that involve the targeting of peers, often repetitively, to create or maintain an imbalance of power (Volk et al., 2014), bullying remains common (Hong & Espelage, 2012) and one of the most damaging youth behaviors (Gladden et al., 2014). Victimization in youth has been shown to lead to commonly occurring psychopathology, such as anxiety (Siegel et al., 2009), depression (Klomek et al., 2008), and suicidality (Takizawa et al., 2014), making the need to understand how to reduce perpetration an important goal. However, how to treat bullying and other destructive peer behaviors in youth, in conjunction with other types of commonly occurring psychopathology, has not been well explicated. Peer relationship behaviors in youth (e.g., bully perpetration, victimization, generalized aggression) have shared relationships to many symptoms of psychopathology (Parker et al., 2015; Prinstein & Giletta, 2016); therefore, evaluating how common childhood symptoms interact with aggressive peer behaviors may provide valuable insights in guiding treatment and in designing interventions.

Anxiety is the most common diagnosis in pediatric samples (Kessler et al., 2005), is marked by deviations in many executive functions (Shanmugan et al., 2016), and presents symptomatically as excessive worry (Weisberg, 2009). Irritability, defined by excessive anger and temper outbursts (Leibenluft, 2017), is a common transdiagnostic symptom in childhood and adolescence (Copeland et al., 2015). Irritability has been consistently linked with many prevalent childhood emotional problems, including internalizing syndromes, like pediatric anxiety (Stoddard et al., 2014), and externalizing problems, including conduct and oppositional defiant disorder (Evans et al., 2017; Humphreys et al., 2019), though internalizing and externalizing syndromes are often not strongly associated with one another (Juvonen et al., 2003). While anxiety is often not significantly related to perpetration (Perino et al., 2019), irritability is often related to perpetration and other aggressive behaviors (Chen et al., 2021; Humphreys et al., 2019). Intriguingly, recent work examining the relationship between anxiety, irritability, and bullying in early adolescence found that anxiety, while not correlated with perpetration, significantly moderated the relationship between adolescent irritability and perpetration. The link between irritability and bully perpetration decreased as anxiety increased (Chen et al., 2021), suggesting that decreases in anxiety, without co-occurring reductions in irritability, could theoretically lead to increases in adolescent perpetration behaviors.

Uncovering why anxiety could impact links between irritability and aggressive behaviors could influence treatment decisions for adolescents presenting with certain symptoms. One potential explanation previously put forth is that bully perpetration behaviors are reflective of an antisocial interpersonal strategy (Juvonen & Ho, 2008), where youth look for opportunities to gain resources at the expense of other peers (Vaillancourt et al., 2003). Perpetration is linked to being viewed as a leader by one’s peers (Vaillancourt et al., 2003), increased social status (Hawley et al., 2007; Rose et al., 2004), intact socio-emotional intelligence (Garandeau & Lansu, 2019; Kaukiainen et al., 1999), and lower emotional distress (Juvonen et al., 2003). Developmentally, adolescence is a period of time where youth increase approach-oriented behaviors (McCormick & Telzer, 2017) and preferentially respond to rewards (Galván, 2013), particularly social ones (Perino et al., 2016; Somerville et al., 2010). If bully perpetration reflects an “approach” oriented behavioral strategy (Kokkinos et al., 2016) used to gain certain types of social resources or advantages (Perino et al., 2019; Vaillancourt et al., 2003; Volk et al., 2012), then increasing levels of anxiety, implicated in behavioral inhibition (Vervoort et al., 2010), may reduce perpetration when all other symptoms are held constant. In short, bully perpetration behaviors may be thought of as approach behaviors used to meet social needs (Hawley, 2002, 2003a; Hawley, 2003b; Hawley, 2015); therefore, symptoms which impact approach motivation systems may paradoxically decrease the relationship between co-occurring psychopathology and perpetration.

While the findings described above are suggestive, there are open questions about the relations among anxiety, irritability, and adolescent aggression. Given the emphasis on bullying as a social strategy in the literature, it is possible that any moderating effect of anxiety may be specific to social anxiety symptoms, rather than generalized anxiety (Chen et al., 2021). Replicating the original moderation and clarifying whether social anxiety is the specific driver is of great import. Additionally, bully perpetration is just one form of aggressive behaviors seen in peer relationships, so clarifying whether anxiety moderates the relationship between only irritability and bullying, or impacts other aggressive peer behaviors, such as generalized aggression, is needed. Significant factor analytic work (Espelage & Holt, 2001) has shown that bullying is distinct from other, more generalized forms of aggression, such as fighting and disagreeableness (Espelage et al., 2014, 2018; Hawley et al., 2010). However, the bullying measure (Jarcho et al., 2019) previously used included items related to fighting, which may be more related to generalized aggression (Espelage & Holt, 2001; Hawley et al., 2010; Juvonen et al., 2003; Vaillancourt et al., 2003). Understanding how anxiety impacts associations of irritability with different forms of aggression comprehensively will better elucidate expected outcomes when symptoms change.

In this manuscript, we sought to replicate the finding that anxiety moderates the link between irritability and bully perpetration, while clarifying whether the effect is specific to social anxiety or generalized anxiety. We explored relations between irritability, anxiety symptoms (social, general), and aggressive peer relationship behaviors (bully perpetration, generalized aggression), as well as interaction effects of anxiety on the relationship between irritability and aggression. We hypothesized that anxiety would moderate the relationship between irritability and perpetration, but that the effect was specific to social anxiety, rather than generalized anxiety. Additionally, we attempted to determine whether the moderation effect differed for different types of aggressive childhood behaviors (bully perpetration, generalized aggression). The results of these analyses aim to improve our understanding of how to better intervene, and what to expect when interventions are applied in these commonly co-occurring behavioral problems in youth.

## Methods

### Participants

The sample used in this study was drawn from an ongoing longitudinal study, the Preschool Depression Study, (Luby et al., 2009) conducted at Washington University School of Medicine. Preschool-aged participants (between ages 3 and 6 years) were recruited from primary care facilities and preschools/daycares in the surrounding metropolitan area from pamphlets about “assessing emotional development.” Parents who responded to promotional material were screened by trained research assistants via telephone interview, to recruit child participants (i) with internalizing psychopathology (endorsement of ≥ 2 symptoms of depression), as well as participants (ii) without psychopathology and (iii) with externalizing psychopathology (endorsement of ≥ 2 symptoms of externalizing psychopathology [ADHD, ODD, or CD]). Participants were excluded if there was evidence of (i) chronic medical illnesses, (ii) neurological problems, (iii) pervasive developmental disorders, or (iv) language/cognitive delays that would impact the ability to answer questionnaires. (Luby et al., 2003) Consent and assent was collected from all participants who completed assessments and all protocols were approved by the Washington University Institutional Review Board.

### Measures

#### Assessment of clinical symptoms of anxiety

Upon screening and consent, participants and parents were assessed via semi-structured interviews by Master’s level raters for psychopathology using the Kiddie-Schedule of Affective Disorders – Present and Lifetime Version (KSADS-PL) (Kaufman et al., 2000). The KSADS-PL is a semi-structured clinician-rated assessment derived from interviews with both parents and children to assess psychopathology in youth, which has been shown to be reliable and valid for childhood psychiatric diagnoses (Kaufman et al., 1997). Master’s level raters were trained to reliability by an experienced clinician (J.L) and to ensure reliability, all interviews were audiotaped and calibration was provided on 20% of each raters’ cases. (Luby & Belden, 2008) Participants completed a baseline assessment at age 3–6, and were subsequently invited back to continue completing assessments of cognitive and social skills and psychopathology every 1–2 years (Gaffrey et al., 2013).

To match the protocol of Chen et al., 2021, we focused our analyses on study wave 12, which focused on early adolescence (mean age 12). Social anxiety and generalized anxiety scores were tabulated by summing symptoms of each from the K-SADS module. The Social anxiety score includes six items (e.g., “is your child shy, fearful in social situations, or uncomfortable with people they don’t know well”) which were marked as either present or not present, while the generalized anxiety score includes nine items (e.g., “does your child worry, have somatic complaints, or have over concern about competence”) which were also marked as present or not present. Positive endorsements were added to create sum scores for social anxiety and generalized anxiety.

#### Irritability

Consistent with the assessment of anxiety, we utilized measures from study wave 12 to assess for irritability. Specifically, we used an irritability measure previously validated within the sample (Vogel et al., 2019) that took a factor analytic approach using items from the clinician-administered Preschool Age Psychiatric Assessment (PAPA) (Egger et al., 1999) (and later the Childhood and Adolescent Psychiatric Assessment:CAPA) (Angold et al., 1995) to differentiate irritability from other forms of emotion dysregulation. Irritability items were from the depression, mania, and conduct modules of the PAPA and CAPA, and included items pertaining to irritability intensity, frequency, spontaneity, irritability concern to caretakers, tearful and crying, angry or resentful intensity and temper tantrum intensity.

#### Bullying behaviors

Bully role behaviors were assessed using a fourteen item composite measure derived from the Parent Report of the Health and Behavior Questionnaire (Essex et al., 2002). Specifically, we conceptualized bullying behaviors as done in the Illinois Bully Scale (Espelage & Holt, 2001) and separately assessed for bully perpetration, generalized aggression, and victimization. The perpetration factor (*α* = .73) consisted of five items (e.g., taunts and teases peers; is cruel, bullies, is mean to others) and each item was rated on a 0–2 scale; the generalized aggression factor (*α* = .68) consisted of 5 items (e.g., temper tantrums; kicks, bites, or hits other children, gets in many fights) and each item was rated on a 0–2 scale; and the victimization factor (*α* = .83) consisted of four items (e.g., is actively picked on; is teased and ridiculed) and each item was rated on a 1–4 scale.

#### Analytic approach

To characterize the sample, we explored relationships between our demographic, clinical (social anxiety, generalized anxiety, irritability), and bully role (bully perpetration, generalized aggression, victimization) continuous variables. We focused exclusively on the behavioral and clinical assessments at the timepoint that most closely matched the sample from Chen et al., 2021, which was (Timepoint 12 [T12]). A total of 169 participants at T12 were assessed for psychopathology and social behaviors in the current analysis. To reduce biases introduced by including missing data in analyses (Woods et al., 2021), we imputed missing data using linear regression (5 iterations) using all the variables included in our analyses (age, sex, race, bullying behaviors, anxiety scores). Descriptive statistics for original and imputed variables are shown in Table 1.

First, we ran zero-order correlations between our variables, to assess primary relationships between psychopathology and bully role behaviors. As an additional step, we ran independent t-tests to determine if sex was significantly related to the aforementioned continuous variables. Next, we used regression-based moderation analyses to explore whether anxiety symptoms were significant moderators of the relationship between irritability and bully perpetration and the relationship between irritability and victimization, while controlling for age, sex, and race. We also aimed to expand upon the interactions explored in Chen et al., 2021 by exploring whether there was a differential interaction when looking at the relationship between irritability and generalized aggression. To graphically explore interactions, we ran Johnson–Neyman tests (Johnson & Neyman, 1936) to determine data ranges where anxiety significantly moderates the relationships between irritability and aggressive behaviors. Finally, given that bully role behaviors (perpetration, generalized aggression, victimization) are correlated and do not appear in isolation, we ran additional interaction models to control for the shared variance between bullying behaviors and more clearly extrapolate relationship between irritability and specific types of aggressive behaviors. We again ran Johnson–Neyman tests (Johnson & Neyman, 1936) to determine data ranges where anxiety significantly moderates the relationships between irritability and aggressive behaviors.

All descriptive statistics, correlations, t-tests, and imputations were run using SPSS (Version 28.0; IBM SPSS, Armonk, NY). All interactions were completed using the PROCESS macro for SPSS (Hayes, 2016). Johnson–Neyman statistics and graphics were run using the Interactions R Toolkit (Long, 2019).

## Results

### Relationships between demographic, clinical, and bully role behaviors

Bully role behaviors were significantly correlated to clinical variables, consistent with past research. Perpetration was positively related with irritability (*r* = .403, *p* < .001) but negatively correlated with social anxiety (*r* = −.185, *p* = .016) and unrelated to generalized anxiety (*r* = −.021, *p* = .791). Generalized aggression was positively correlated with irritability (*r* = .535, *p* < .001) but not significantly correlated with social (*r* = .013, *p* = .869) or generalized (*r* = .145, *p* = .059) anxiety. Irritability was significantly correlated with generalized anxiety (*r* = .366, *p* < .001) but not social anxiety (*r* = .046, *p* = 552, see Table 2 for full results and supplemental Figure 1A–C to see distribution of bully role behaviors). When running independent samples t-tests, generalized aggression (*t*(167) = 2.223, *p* = .028) and irritability (*t*(167) = 2.190, *p* = .030) were the only variables with significant relations to sex; for both generalized aggression (male mean = 0.23, *SD* = 0.26; female mean = 0.15, *SD* = 0.20), and irritability (male mean = 39.88, SD = 8.26, female mean = 37.35, *SD* = 6.56) males scored higher than females.

### Moderation effects of anxiety on bully role behaviors

**Figure 1.**
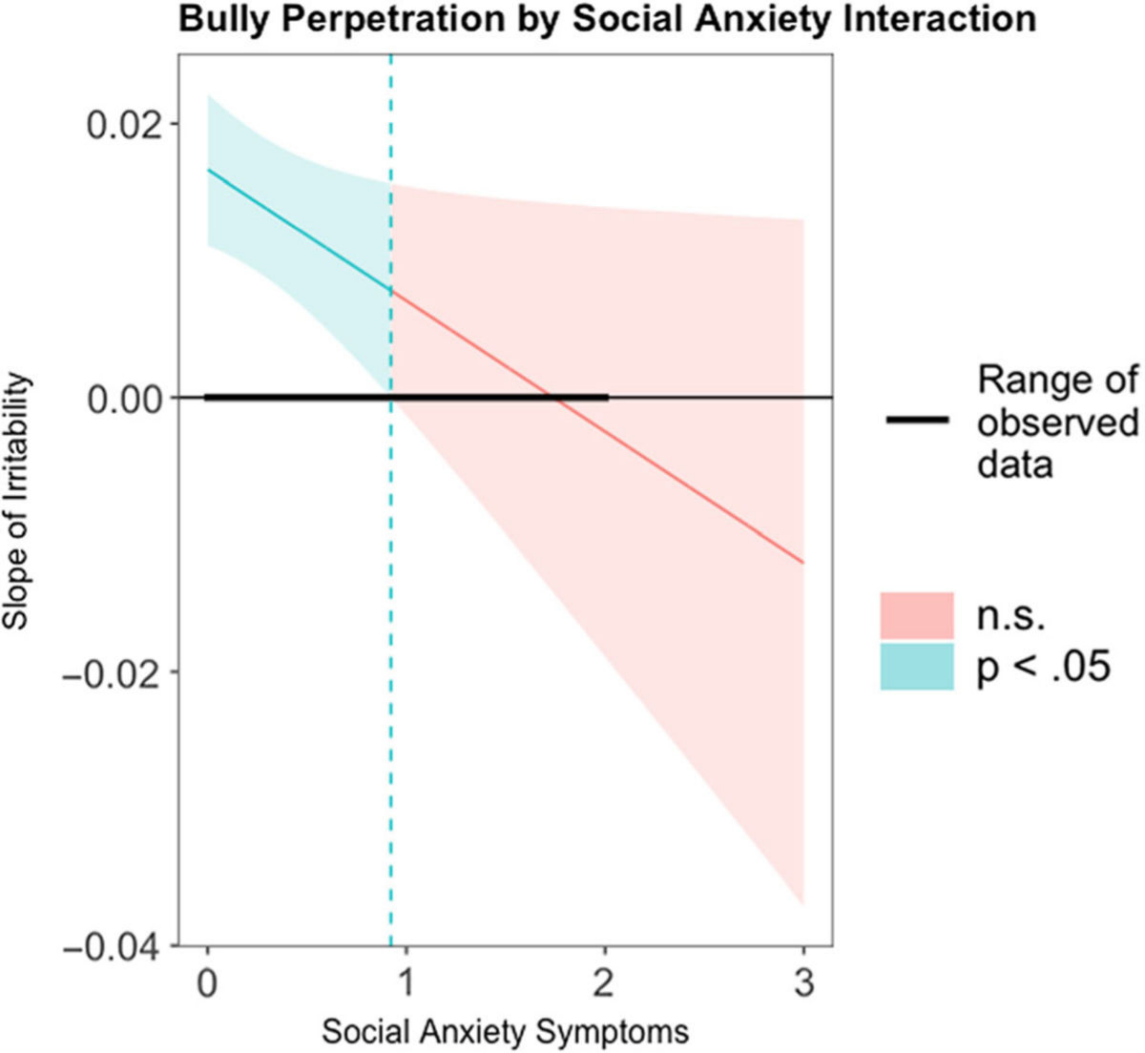
Social anxiety moderates the relationship between irritability and bully perpetration. The Johnson–Neyman technique was employed to explore the moderating effect of social anxiety on the relationships between irritability and bully perpetration, while controlling for demographic variables (age, sex, race). We observed that when social anxiety symptom endorsement was low (below 0.92 symptoms), the association between irritability and perpetration was significant and positive. As social anxiety increased, we saw a corresponding reduction to nonsignificance in the association between irritability and perpetration.

Our first two regression-based moderation analyses explored whether social anxiety or generalized anxiety significantly moderated the relationship between irritability and bully perpetration. As we hypothesized, there was a significant interaction term between irritability and social anxiety (*t*(162) = −2.11, *b* = −.0096, *p* = .036, Δ*R*^*2*^ = .0198, *F*(1, 162) = 4.465), such that as social anxiety increased, the association between irritability and bully perpetration decreased. Using the Johnson–Neyman method, we observed that there was a significant positive association between irritability and bully perpetration at low levels of social anxiety, but no association between irritability and bully perpetration at higher levels of social anxiety. Specifically, irritability was positively, significantly associated with levels of perpetration when there were less than 0.92 social anxiety symptoms (CI [.000, .0157], *p* = .050, see Fig. 1).

When we ran our regression-based moderation analysis using generalized anxiety symptoms instead of social anxiety symptoms, we observed that there was no significant interaction term (*t*(162) = .22, *b* = .0006, *p* = .822, Δ*R*^*2*^ = .0006, *F*(1, 162) = 0.051). Additional moderation analyses exploring whether anxiety moderated the relationship between irritability and victimization were nonsignificant for both social anxiety (*t*(162) = .049, *b* = .0005, *p* = .961, Δ*R*^*2*^ = 0, *F*(1, 162) = 0.0024) and generalized anxiety (*t*(162) = .581, *b* = .0035, *p* = .561, Δ*R*^*2*^ = 0018, *F*(1, 162) = 0.338).

Next, we explored whether generalized aggression was similar to bully perpetration in that it was moderated by anxiety. However, we found that neither social anxiety (*t*(162) = .27, *b* = .0010, *p* = .789, Δ*R*^*2*^ = .0003, *F*(1, 162) = 0.072) nor generalized anxiety (*t*(162) = .28, *b* = .0006, *p* = .777, Δ*R*^*2*^ = .0003, *F*(1, 162) = 0.080) significantly moderated the relationship between irritability and generalized aggression.

**Figure 2.**
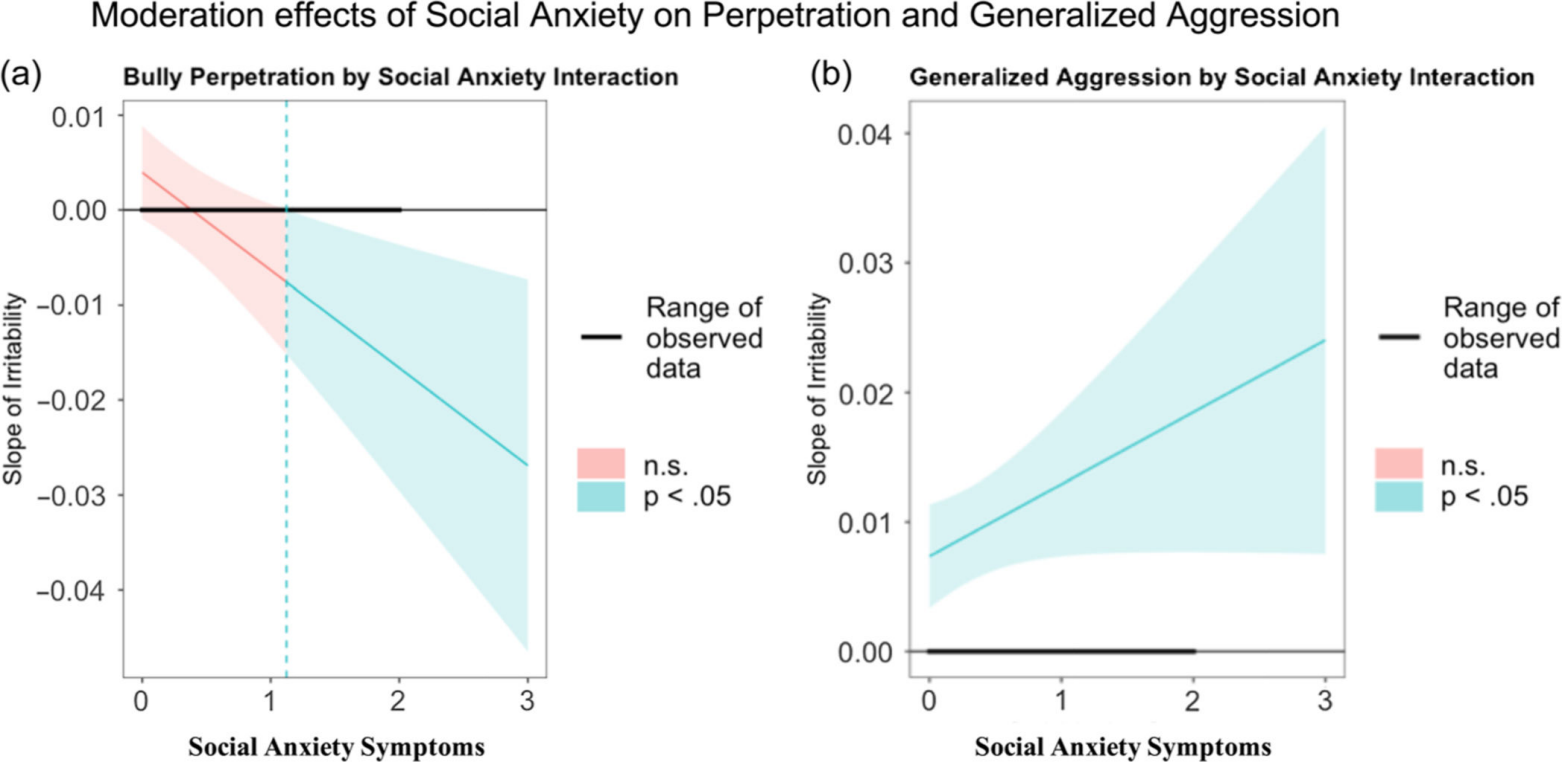
Moderation effects of social anxiety between irritability and forms of aggressive behaviors. The Johnson–Neyman technique was employed to explore the moderating effect of social anxiety on the relationships between **(*a*)** irritability and bully perpetration, and **(*b*)** irritability and generalized aggression, while controlling for other bully role behaviors and demographic variables. For bully perpetration, we saw a nonsignificant negative effect when social anxiety endorsement was low, and a significant, negative effect when social anxiety endorsement was greater than or equal to 1.12 symptoms (CI [−.08, .0]). Increasing social anxiety corresponded with a negative relationship between irritability and bully perpetration. For generalized aggression, there was no statistical significance transition point, as the relationship between irritability and generalized aggression was the same regardless of the level of social anxiety.

Given that only social anxiety, and not generalized anxiety, significantly moderated the relationship between irritability and bully behaviors, we focused solely on social anxiety in our follow-up set of moderation analyses. When examining the link between irritability and bully perpetration, while additionally controlling for generalized aggression and victimization, the moderation effect of social anxiety was still significant (*t*(160) = −2.94, *b* = −.01, *p* = .0038, Δ*R*^*2*^ = .0229, *F*(1, 160) = 8.635); specifically, irritability was now significantly negatively associated with perpetration when there was endorsement of more than 1.12 social anxiety symptoms (see Fig. 2a). Social anxiety still did not significantly moderate the link between irritability and generalized aggression (*t*(160) = 1.85, *b* = .0056, *p* = .065, Δ*R*^*2*^ = .009, *F*(1, 160) = 3.429) when additionally controlling for bully perpetration and victimization. For illustrative purposes, we provide the Johnson–Neyman plot showing that the link between irritability and generalized aggression is significantly positive at all levels of social anxiety (see Fig. 2b).

## Discussion

The current study found that as social anxiety symptoms increased, the link between irritability and bully perpetration became more negative. Without accounting for other bully role behaviors, we observed that as adolescents’ endorsement of social anxiety symptoms increased, the link between irritability and bully perpetration decreased and became nonsignificant. When accounting for other bully role behaviors (generalized aggression, victimization), we observed that as there was greater endorsement of social anxiety symptoms, the association between irritability and bully perpetration actually became significantly negative. The moderating of anxiety on the link between irritability and bully perpetration was seen with social anxiety – but not generalized anxiety – and these effects on the relationship between irritability to bully role behaviors was specific to bully perpetration, and not generalized aggression nor victimization. Bully perpetration was negatively related to social anxiety but positively related to irritability. Irritability was positively related to both generalized aggression and victimization; however, neither generalized aggression nor victimization was related to anxiety nor did anxiety moderate the link between irritability and these behaviors. Our results suggest that symptoms of psychopathology have complex associations with bully role behaviors, and that changes in one domain of psychopathology could impact the manifestation of a broad set of social behaviors.

We replicated some, but not all of the findings previously reported by Chen et al. (2021). We also found a significant relationship between irritability and victimization and found that anxiety did not moderate the link between irritability and victimization. We did not find that generalized anxiety significantly moderated the relationship between irritability and perpetration (Chen et al., 2021); however, we did find that social anxiety symptoms moderated this relationship. Chen et al. (2021) did not dissociate whether particular types of anxiety were driving moderation effects, so it is unclear if social anxiety was also responsible for the moderation they observed. The observation that social anxiety significantly negatively moderates the link between irritability, a transdiagnostic symptom (Klein et al., 2021), and perpetration to the point where perpetration becomes negatively associated with irritability is quite interesting. It suggests that even though irritability predicts perpetration, the presence of social anxiety may blunt this expression.

Such a finding is consistent with the hypothesis that bully perpetration reflects a social strategy, and that symptoms which reduce social approach behaviors (e.g., social anxiety) may reduce expected links between psychopathology (such as irritability) and perpetration (Thomas et al., 2018). Bully perpetration has been shown to confer certain types of social advantages, such as increased numbers of romantic partners (Provenzano et al., 2018; Volk et al., 2015), winning competitive endeavors (Dane et al., 2022), gaining social status (Spadafora et al., 2022), and deterring rivals (Cairns et al., 1988). While irritability is related to aggression writ large (Humphreys et al., 2019), bullying is unique in that it is targeted, goal-directed and inherently about social position (Volk et al., 2014). We posit that social anxiety acts as an inhibitory force, and when irritability is kept constant, increasing social anxiety will lead to reduced perpetration. On the other hand, other aggressive behaviors likely stem from frustration-intolerance or impulse control difficulties and not social goals (Little et al., 2003). As expected, social anxiety did not moderate the association between irritability and other aggressive behaviors, which were positively associated with irritability at all levels of social anxiety. Therefore, if irritability is held constant, increasing social anxiety may blunt the link between irritability and perpetration but remain positively associated with generalized aggression.

Further explicating how changes in symptom levels may impact the ecology of peer-networks broadly, and bully perpetration specifically, may be an important factor to consider when evaluating treatment efficacy (Gaffney et al., 2021). Addressing psychopathology in individuals may lead to positive, measurable effects for some behaviors, while counter-intuitively creating negative effects in others, suggesting a need for researchers to comprehensively assess psychopathology and social behaviors rather than focus on individual syndromes or behavioral phenotypes. While treating social anxiety in adolescents will have myriad positive outcomes (Fisher et al., 2004), it is imperative to also address underlying irritability, lest improvements in one domain (anxiety) potentially lead to decrements in another (less prosocial behaviors). Additionally, given the link of irritability to other adverse social behaviors (e.g., victimization), understanding whether interventions have impacts across a wide variety of domains is imperative. Furthermore, bully perpetration behaviors are heterogenous (Farrell et al., 2014) and evolving (Waasdorp et al., 2017) so further research is necessary to determine whether the moderating effect of social anxiety on linkages between irritability and bully perpetration are universal or differentially impact specific bullying behaviors. For example, bullying that doesn’t require an audience (with a romantic partner) or cyberbullying, which may provide anonymity, may be less impacted by social anxiety compared to relational perpetration. The answers to these questions may help determine how to best target individual-level interventions based on behavioral phenotype.

This study needs to be considered in light of its limitations. The recruitment and assessment protocols used in this longitudinal study (Luby et al., 2009) resulted in their being ample participants with symptoms of psychopathology. However, our measures of anxiety (clinician-rated symptoms) (Kaufman et al., 2000) and bully role behaviors (composite measure) (Essex et al., 2002) would have benefitted by utilizing alternative information sources, such as peer reports and self-reports (Makol et al., 2020). It is currently unclear if the relations reported here would equally apply to all forms of bullying. For example, cyberbullying, which often requires less direct contact and may provide perpetrators with anonymity and physical distance from victims may demonstrate weaker links with social anxiety. This hypothesis was not testable in our dataset, but is worthy of further inquiry. Additionally, given the relatively small distribution of scores in our measures of anxiety, using measures with greater distribution across trait levels may help improve statistical assessments. To increase the precision of our moderation analyses examining the links between irritability and bully perpetration, we controlled for demographic variables and co-occuring bully role behaviors. We observed that social anxiety interaction effect accounted for 2% of the model variance, which would be considered a small effect. While our own findings partially replicate prior work, the need to replicate these effects with higher powered samples is paramount. While this work adds vital information by examining generalized aggression in addition to perpetration, it is important for future work to comprehensively assess how associations with psychopathology relate to other types of bully role behaviors, such as prosocial behavior (i.e., bystander intervention) (Jenkins et al., 2021). We hypothesize that increasing social anxiety may moderate the relationship between irritability and other approach-oriented behaviors, be they antisocial or prosocial, and suggest this as a needed line of inquiry.

This study demonstrates that social anxiety significantly impacts the relationship between irritability and bully perpetration. Bully perpetration is a persistent (Espelage & Swearer, 2003) and damaging (Brimblecombe et al., 2018) problem, and current interventions have positive, albeit small, effects (Fraguas et al., 2021). Treating common symptoms can impact the relationship between perpetration and other related symptoms, which may ultimately provide informative insight into how symptom change can have cascading effects on reducing various forms of antisocial behavior.

## Supplementary Material

1

## Figures and Tables

**Table 1.** Descriptive statistics of demographic, clinical and behavioral variables

Sample descriptive statistics								

Original data					Imputed Data			
	
Demographic Variables	N	Mean	SD	Range	N	Mean	SD	Range

Sex								

Female	81				–			

Male	88				–			

Race								

White	73				74			

Black	69				75			

Asian/Native	20				20			

American/More than one								

race								

Age	169	12.42	0.94	9.17–14.89				

Clinician Measures	N	Mean	SD	Range	N	Mean	SD	Range

Social Anxiety Symptoms	162	0.12	0.392	0–2	169	0.12	.396	0–2

Generalized Anxiety Symptoms	162	0.15	0.752	0–6	169	0.17	.745	0–6

Parent Reported Measures	N	Mean	SD	Range	N	Mean	SD	Range

Bully Perpetration	125	0.19	0.302	0–1.40	169	0.24	.283	0–1.40

Generalized Aggression	125	0.16	0.266	0–1.40	169	0.19	.240	0–1.40

Victimization	125	1.40	0.627	1–4.00	169	1.50	.584	1–4.00

Irritability	125	37.48	8.197	31.48–67.05	169	38.67	7.58	31.48–67.05

**Table 2.** Correlation matrix for demographic, clinical and behavioral variables

Variables	1	2	3	4	5	6	7
1. Irritability							
2. Social Phobia	.046						
3. GAD	.366***	.434***					
4. Perpetration	.403***	−.185*	−.021				
5. Aggression	.535***	.013	.145	.676***			
6. Victimization	.326**	.022	.094	.445***	.410***		
7. Age	−.094	.118	.082	−.139	.001	−.130	

1. Irritability, 2. Number of Social Phobia Symptoms, 3. Number of Generalized Anxiety Symptoms, 4. Bullying Perpetration, 5. Generalized Aggression, 6. Bullying Victimization, 7. Age.

* *p*<.05

** *p* < .01

*** *p* < .001.
