# Supplementary material for: Social anxiety moderates the association between adolescent irritability and bully perpetration
Source: Dev Psychopathol. Author manuscript; Available in PMC 2025 Nov 1. (PMC11393177; doi:10.1017/S0954579424000439)
Supplement: 1 [file NIHMS1965643-supplement-1.docx]

Supplemental Figure 1. A-C

Histograms showing the distribution of bully role behaviors, including bully perpetration (A), generalized aggression (B), and victimization behaviors (C)) are shown below.

**A**

**B**

**C**
